# Metabolic Improvement and Weight Reduction After Large Endoscopic Submucosal Dissection Including the Papilla for Duodenal Adenoma: A Case Report

**DOI:** 10.1002/deo2.70433

**Published:** 2026-09-17

**Authors:** Chi‐Chu Lo, Chen‐Shuan Chung, Wei‐Wei Lee, Chien‐Chen Tsai, Kurato Miyazaki, Motohiko Kato

**Affiliations:** ^1^ Department of Internal Medicine Division of Gastroenterology and Hepatology, Far Eastern Memorial Hospital New Taipei City Taiwan; ^2^ Department of Electrical Engineering, Yuan Ze University Taoyuan Taiwan; ^3^ Taiwan Association for the Study of Intestinal Diseases (TASID) Taoyuan Taiwan; ^4^ Department of Anatomic Pathology Far Eastern Memorial Hospital New Taipei City Taiwan; ^5^ Center For Diagnostic and Therapeutic Endoscopy Keio University School of Medicine Tokyo Japan

**Keywords:** adenoma, duodenum, endoscopic submucosal dissection, obesity, weight loss

## Abstract

Endoscopic submucosal dissection (ESD) has become an established therapeutic approach for duodenal adenomas, although it is technically demanding. Duodenal mucosal resurfacing (DMR) is an emerging endoscopic metabolic therapy designed to ablate and regenerate duodenal mucosa. To date, no reports have described ESD including the papilla (ESDIP) producing similar metabolic effects to DMR. A 36‐year‐old Asian man with an elevated body mass index of 30.16 kg/m^2^ was diagnosed with a large duodenal laterally spreading tumor, measuring 80 × 70 mm in size and extending to the major papilla. Water‐pressure method‐assisted ESDIP was performed with en bloc resection, and histopathology demonstrated a tubulovillous adenoma with low‐grade dysplasia and uninvolved margins. Remarkably, during the 2‐ and 8‐week follow‐up periods, the patient exhibited reductions in total body weight of 5.9% and 7.9%, respectively, and a marked improvement in glycemic control, with fasting blood glucose decreasing from 210 to 113 mg/dL despite no changes in lifestyle or medication. These metabolic improvements suggest a possible clinical overlap between extensive duodenal mucosal resection via ESDIP and DMR. However, given the limitations of a single case, these findings warrant cautious interpretation and further study to clarify the underlying mechanisms.

## 1 Introduction

Obesity is a chronic, relapsing, and progressive disease characterized by an excessive accumulation of adipose tissue, which is associated with several comorbidities. In adults, it is commonly defined as a body mass index (BMI) of 30 kg/m^2^ or higher, while this diagnostic threshold is generally lower in Asian populations (≥27.5 kg/m^2^) [1]. Management of obesity typically includes monitoring body weight and controlling blood pressure, glucose levels, lipid profiles, and other concurrent metabolic disorders [1]. Duodenal mucosal resurfacing (DMR) is a promising endoscopic bariatric and metabolic therapy (EBMT) that aims to enhance insulin sensitivity and promote weight loss by ablating and regenerating the duodenal mucosa [2, 3]. Although well‐established for treating superficial duodenal neoplasia, endoscopic submucosal dissection (ESD) remains technically challenging with high complication rates due to the thin duodenal wall and complex anatomy [4]. To date, no studies have reported that ESD involving the papilla (ESDIP) induces metabolic improvements comparable to those observed with DMR.

## 2 Case Report

**FIGURE 1:**
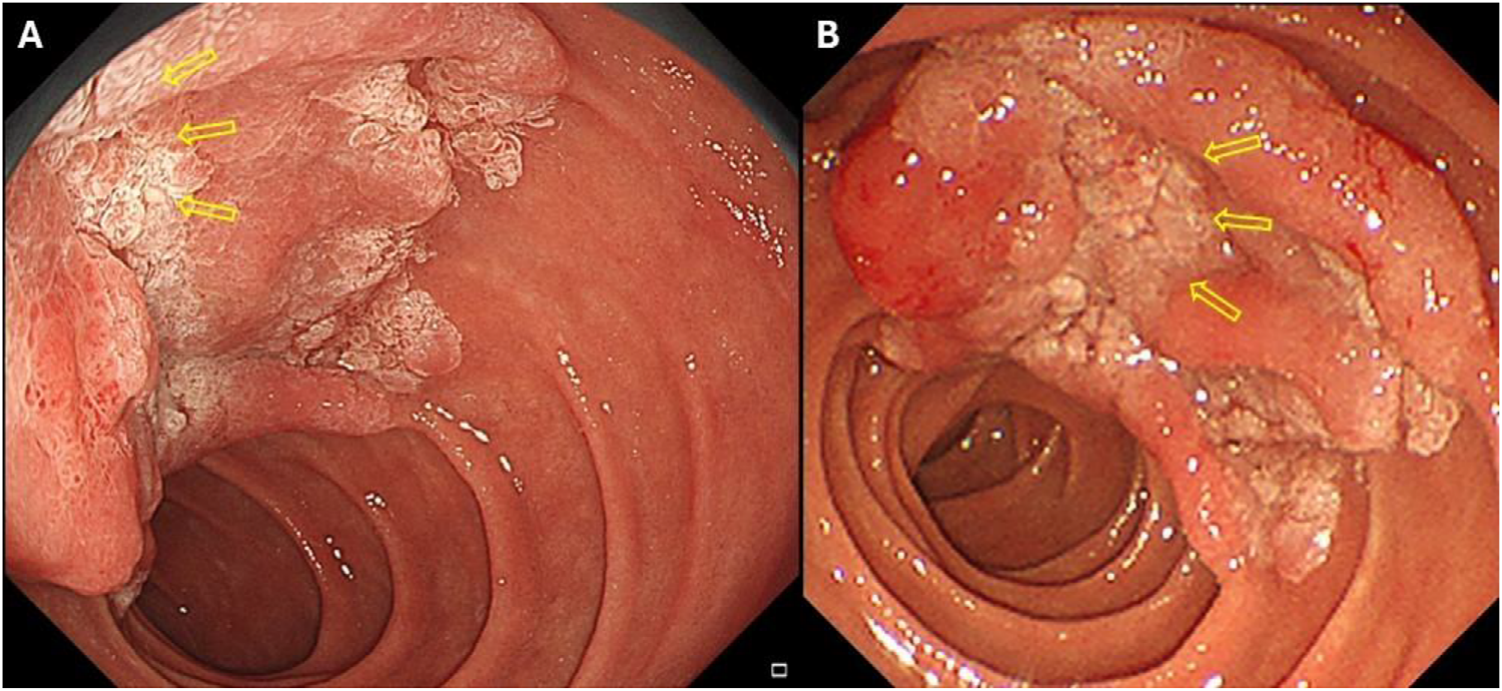
(A, B) Large duodenal laterally spreading tumor extending to the major papilla. Yellow arrows point to the major papilla.

**FIGURE 2:**
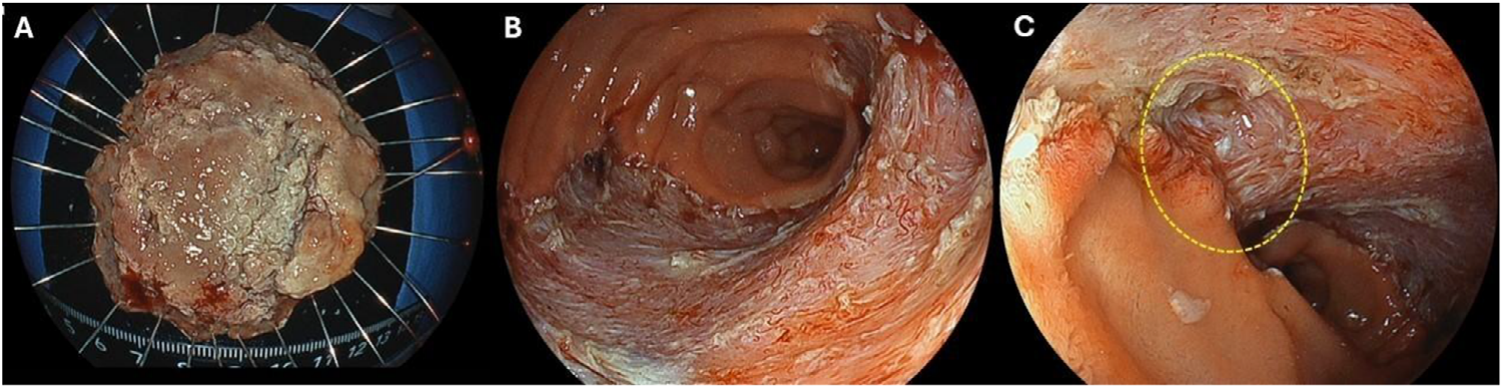
(A) Specimen after endoscopic submucosal dissection including the papilla. (ESDIP). (B) Post‐ESDIP wound involving over 50% of the circumference. (C) Post‐ESDIP wound maintaining the structure and patency of the pancreatic and bile duct (circled in a dashed yellow line).

**FIGURE 3:**
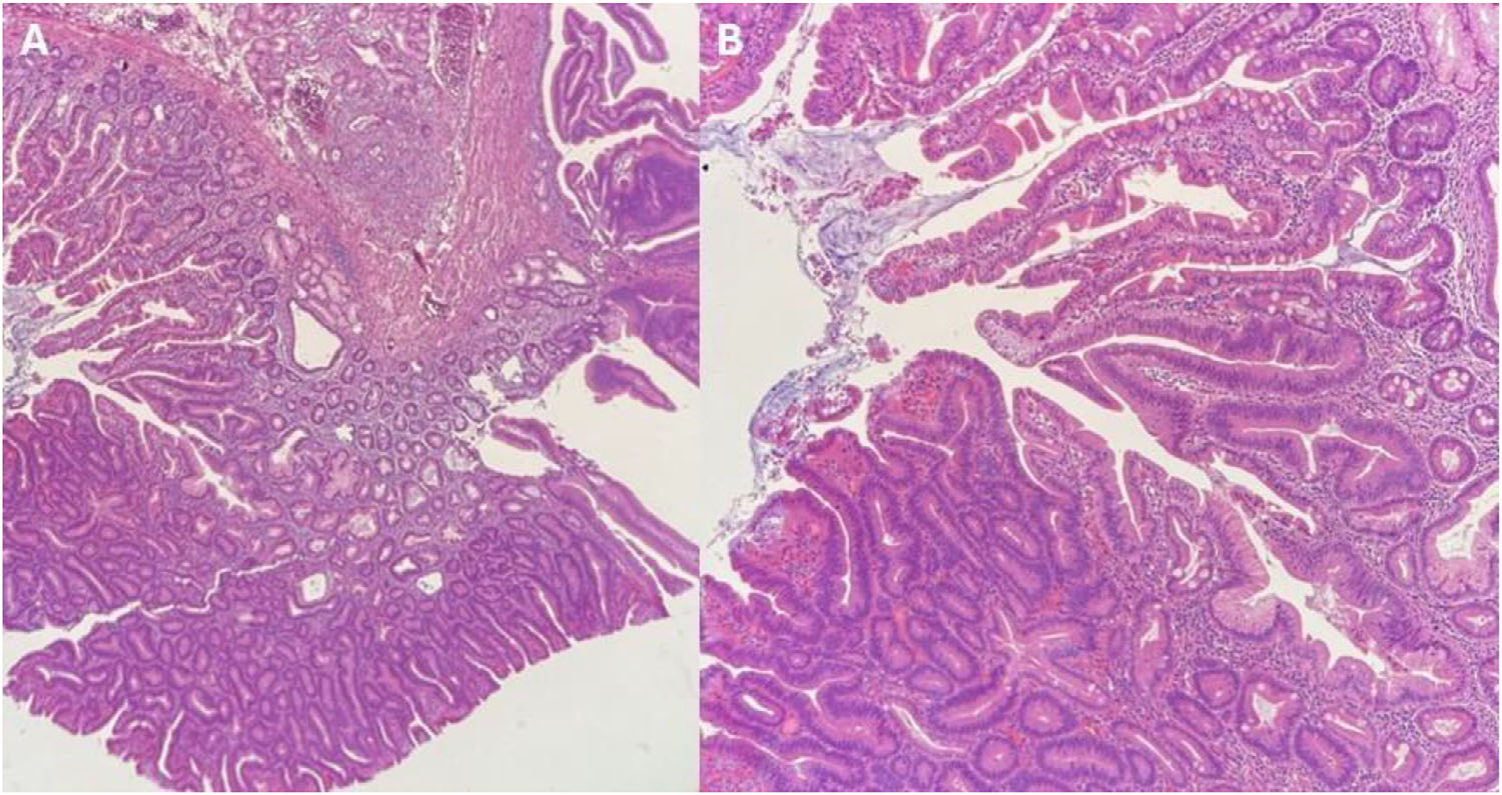
Tubulovillous adenoma with low‐grade dysplasia of the duodenal papilla. (A) Hematoxylin and eosin stain, 40× magnification. (B) Hematoxylin and eosin stain, 100× magnification.

**FIGURE 4:**
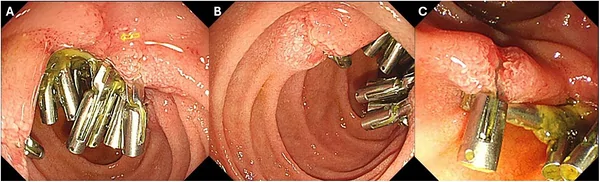
(A) Post‐ESDIP wound with hemoclips in situ and surrounding ulceration (one yellow arrow pointing at the ulceration). (B) The gastroscope was passable without resistance. (C) The papillary orifice was patent.

We report a 36‐year‐old obese Asian man (183 cm in height, 101 kg in weight, BMI 30.16 kg/m^2^) diagnosed with a large duodenal laterally spreading tumor (LST), measuring 80 mm × 70 mm and extending to the major papilla (Figure 1, VIDEO S1). Endoscopic ultrasound and magnetic resonance imaging confirmed no invasion of the main pancreatic duct or common bile duct. Water‐pressure method‐assisted ESDIP was performed using a therapeutic gastroscope (EG‐840T; Fujifilm Corp., Tokyo, Japan) equipped with a water‐jet function (JW‐3 water pump; Fujifilm Corp., Tokyo, Japan). The underwater method with instillation of normal saline was applied to optimize visualization and maintain mucosal lifting by buoyancy. Without mucosal marking, a submucosal injection (a mixture of glycerol solution and indigo carmine) was administered to achieve adequate lifting. A mucosal incision was made initially at the anal site, followed by an oral site mucosal incision and stepwise submucosal dissection using an electrosurgical knife (Dual‐J knife [KD‐655L]; Olympus Medical Systems Corp., Tokyo, Japan) with the setting of VIO 3 electrosurgical generator (ERBE Elektromedizin GmbH, Tübingen, Germany) in EndoCut‐I 1‐2‐2 for cutting and Precisect 3.3 for dissection. Special attention was given to the region of the major papilla to ensure en bloc resection while preserving the integrity of the pancreatic and bile ducts. The specimen (Figure 2A) was retrieved en bloc for histopathological evaluation, and the post‐ESDIP wound was shown in Figure 2B,C and VIDEO S2. Following ESDIP, endoscopic retrograde cholangiopancreatography was performed, and plastic stents were placed in both the common bile duct and the main pancreatic duct. On the day following a large‐area ESDIP of the duodenal LST, the patient developed mild epigastric pain accompanied by an elevated serum lipase level of 482 U/L (normal range, 11–82 U/L). After fasting for three days, the abdominal pain resolved, and serum pancreatic enzyme levels returned to normal. Histopathological evaluation revealed a tubulovillous adenoma with low‐grade dysplasia and uninvolved margins (Figure 3). He was discharged uneventfully on day 5, and the follow‐up endoscopic findings 4 weeks after ESDIP were shown in Figure 4. He decreased his weight from 101 to 95 kg (−5.9%) at the 2‐week follow‐up. Furthermore, his weight loss persisted after discharge despite resuming a normal diet, with body weight decreasing to 93 kg 8 weeks after the procedure (a 7.9% reduction from baseline). In addition, the fasting blood glucose level decreased from 210 to 113 mg/dL at 2 weeks, despite no changes in lifestyle or medication.

## 3 Discussion

Between 1990 and 2021, global adult obesity prevalence surged dramatically — rising 104.9% in females and 155.1% in males [5]. In 2021, when over one billion adults of each sex were classified as having either overweight (BMI 25–<30) or obesity (BMI ≥30), the global prevalence of overweight and obesity peaked at approximately age 50 for both males and females [5]. By 2050, individuals aged 25 and over with overweight or obesity are projected to reach 3.8 billion under this trend [5]. Obesity is a chronic, relapsing disease marked by excess adiposity and altered organ function (clinical obesity), typically assessed by BMI or waist circumference [1]. Intervening during preclinical obesity—before organ dysfunction—is crucial to prevent progression and complications [1]. Current therapeutic strategies for management include lifestyle modifications, nutrient‐stimulated hormone therapies, and EBMTs [6, 7]. EBMT is generally divided into gastric and small‐bowel categories, both of which are effective options for obesity or glucose intolerance [7]. DMR is one of the novel endoscopic techniques that have emerged over the past decade. A single session of DMR involves inserting a flexible endoscope and positioning a balloon catheter in the proximal duodenum. After inflating the balloon and injecting saline for a mucosal lift, thermal ablation is performed using a combination of hot and cold water. The total ablation length of DMR was around 9–10 cm distal to the Ampulla of Vater towards the Ligament of Treitz. This procedure is hypothesized to rejuvenate and repair the mucosal hyperplasia of the duodenum, thereby restoring hormonal and metabolic homeostasis and reducing insulin resistance and obesity [2]. The safety and clinical feasibility of this procedure were rigorously evaluated in the REVITA‐2 trial, a double‐blind, multicenter, sham‐controlled study, which showed that DMR achieved high technical success and a favorable safety profile [3]. In terms of clinical efficacy in the REVITA‐2 trial, the DMR group demonstrated short‐term improvements compared to sham group in HbA1c control at 6 months (−14.2 vs. −4.4 mmol/mol, *p* = 0.002) when baseline fasting plasma glucose ≥ 10 mmol/L. Also, in the European population arm, the median weight reduction 6 months post‐DMR was significantly greater than in the sham group (−2.4 vs. −1.4 kg, *p* = 0.012) [3].

The prevalence of superficial non‐ampullary epithelial duodenal tumors ranges between 0.03% and 0.4%, with rising incidence attributed to improved endoscopic techniques [4]. Endoscopic resection is feasible, with ESD indicated for lesions >2 cm or confined to superficial submucosa [4]. Technical difficulty, high intra‐procedural perforation risk, and prolonged procedure time stem from three main factors: sharp angulation (superior/inferior duodenal angles), lesion size >4 cm, or circumferential involvement >50% [8]. Given reported post‐ESD perforation rates of 13%–50%, duodenal ESD remains largely restricted to expert centers requiring careful pre‐procedural assessment [4, 8].

Endoscopic papillectomy (EP) is generally not recommended for lesions > 2 cm and has a local recurrence rate of approximately 10%, which limits its clinical use [9]. ESDIP is indicated for lesions without submucosal invasion, those unsuitable for en bloc EP, and those without confirmed invasion of the pancreatic duct or intrapancreatic bile duct [10]. In 54 duodenal tumors (median size: 39 mm) involving the major or minor papilla, Yahagi et al. reported en bloc and R0 resection rates of 96% and 46%, respectively, with a 15% intraprocedural perforation rate. At 12 months post‐ESDIP, the cumulative local recurrence rate was 15%, and the overall survival rate was 96% [10].

In our case of a large duodenal tumor involving the major papilla, the en bloc specimen measured 80 × 70 mm, with a post‐ESDIP wound involving more than 50% of the lumen circumference and the major papilla. We observed a metabolic response pattern similar to that of DMR in ESDIP, with body weight loss and a reduction in fasting plasma glucose within 2 weeks. However, any shared biological mechanism with DMR remains hypothetical. Limitations of this case include early post‐procedural pancreatitis, temporary fasting, and reduced oral intake as potential confounders of initial weight loss and glycemic improvement, as well as the lack of comprehensive objective obesity testing. Further studies are encouraging the use of duodenal ESD in obese cases and require long‐term analysis of detailed obesity parameters, including BMI, waist circumference, glucagon‐like peptide‐1, ghrelin, HbA1c, homeostatic model assessment of insulin resistance, and metabolic syndrome parameters. These preliminary observations highlight the complexity of duodenal mucosal changes following ESDIP. Given the emerging interest in DMR, further clinical and mechanistic studies are warranted to elucidate whether ESDIP shares overlapping metabolic pathways with DMR and to clarify its potential downstream metabolic effects.

## Author Contributions

**Conceptualization**: Chi‐Chu Lo, Chen‐Shuan Chung, and Motohiko Kato. **Data curation**: Chi‐Chu Lo and Chien‐Chen Tsai. **Methodology**: Wei‐Wei Lee and Chien‐Chen Tsai. **Project administration**: Chen‐Shuan Chung. **Resources**: Chen‐Shuan Chung and Motohiko Kato. **Supervision**: Chen‐Shuan Chung and Kurato Miyazaki. **Writing – original draft preparation**: Chi‐Chu Lo. **Writing – review & editing**: Chi‐Chu Lo, Chen‐Shuan Chung, Wei‐Wei Lee, Kurato Miyazaki, and Motohiko Kato.

## Funding

The authors have nothing to report.

## Ethics Statement

This study was approved by the Institutional Review Board of Far Eastern Memorial Hospital (FEMH‐36262‐T).

## Consent

Informed consent was waived for the patient due to the retrospective collection and deidentification of the information presented in this brief report.

## Conflicts of Interest

The authors declare no conflicts of interest.

## Supporting information




**VIDEO S1**: Large duodenal laterally spreading tumor extending to the major papilla with white opaque substance under magnifying endoscopy with narrow‐band imaging.

**VIDEO S2**: Post‐endoscopic submucosal dissection including the papilla specimen (submucosal side) and wound.
